# Combinational effect of gamma irradiation and sustainable bioactive absorbent bacterial cellulose pads impregnated with tangerine essential oil against some bacterial fish fillet-borne pathogens

**DOI:** 10.1186/s40643-026-01061-0

**Published:** 2026-05-05

**Authors:** Reham M. M. Abdelkader, Doaa. A. Hamed, Hanan H. Abdel-Khalek

**Affiliations:** Radiation Microbiology Department, National Center for Radiation Research and Technology (NCRRT), Egyptian Atomic Energy Authority, Cairo, Egypt

**Keywords:** Food pads, Bacterial cellulose, Hurdle technology, Antimicrobial, Tangerine essential oil, Gamma irradiation, *Klebsiella oxytoca*, *Serratia ficaria*, *Enterobacter cloacae*, *Kocuria rosea*

## Abstract

Antimicrobial food pads play a key role in the food packaging industry by improving food safety and quality. To control the growth of bacterial pathogens in fish fillets, this study aims to develop a bioactive system using absorbent food pads composed of bacterial cellulose (BC) infused with tangerine essential oil (TEO) as an antibacterial agent. The effects of active BC-pads, gamma irradiation, and their combination on artificially inoculated bacteria (previously isolated) were studied for 6 days at 4 °C using fish fillet samples as a model food system. The results revealed that the initial population counts of *Klebsiella oxytoca, Serratia ficaria, Enterobacter cloacae,* and *Kocuria rosea,* were 5.47, 5.63, 5.14, and 5.25 log CFU/g, respectively. These counts increased during the storage period, reaching 8.80 log CFU/g as a mean for all tested strains. After six days of storage, BC, BC/TEO, BC + 1.0 kGy, and BC/TEO + 1.0 kGy pads reduced the initial load of the four bacteria in the fish fillet samples by [1.59, 1.68, 1.92, 2.35], [3.21, 3.12, 3.36, 3.72], [3.53, 3.26, 3.26, 3.37] and [4.97, 5.02, 4.95, 4.83] log CFU/g, respectively. The combination of BC and TEO with low-dose gamma radiation (1.0 kGy) enhances the decontamination effectiveness of fish fillets through synergistic effects. FTIR analysis revealed a slight shift in the absorbance peaks of some functional group interactions and the formation of new bonds between BC and TEO in the presence of gamma irradiation. The study has successfully developed sustainable, eco-friendly, functional bioactive food pads that reduce microbial growth and prevent spoilage of fish fillets during storage.

**Graphical abstract:**

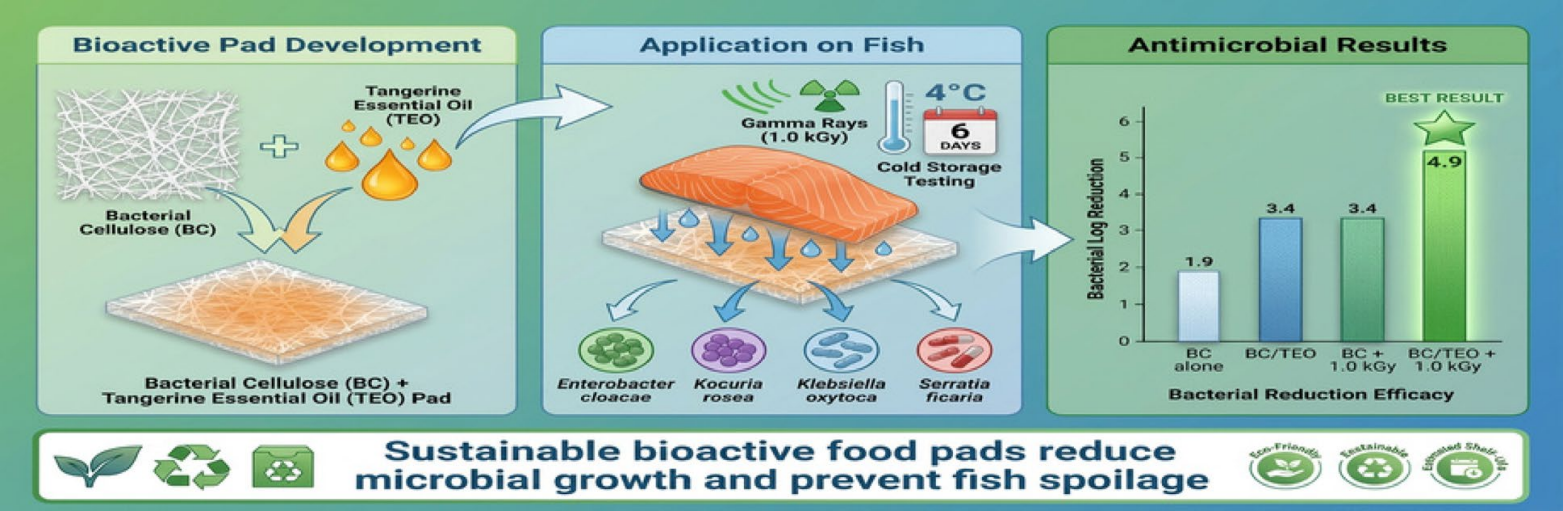

**Supplementary Information:**

The online version contains supplementary material available at 10.1186/s40643-026-01061-0.

## Introduction

Foodborne diseases are considered a worldwide public health problem because of its high morbidity and mortality. Seafood, particularly fish, is a widely consumed nutritious foodstuff, but it is also perishable and easily contaminated (Lerma-Fierro et al. 2020). The deterioration of fish products may result from protein and lipid oxidation, autolysis, the formation of biogenic amines/melanosis, and microbial degradation (Laorenza et al. 2022). There are three categories of pathogenic bacteria that are associated with and transmitted via fish products (Böhme et al. 2011; Pantosti 2012; Elsherief et al. 2014; Odeyemi et al. 2020), the indigenous bacteria are the first group that are distributed in aquatic environments and form part of the natural microbiota of fish (*Aeromonas hydrophila, Clostridium botulinum*, *Listeria monocytogenes, Vibrio cholera,* and *V. parahaemolyticus*). The second group is the non-indigenous or enteric bacteria (*E. coli*, *Salmonella* spp., *Shigella* spp., and *Staphylococcus aureus*) that are present in fish because of fecal contamination of the water where they are captured, cultivated, or derived from poor handling practices. While *Lactobacillus* sp., *Pseudomonas* sp*., Bacillus cereus*, *C. perfringens*, *Proteus* sp., and *Shewanella* sp. represent the third group of bacteria that contaminate fish via improper processing, storage, or cooking (Lerma-Fierro et al. 2020; Thaotumpitak et al. 2022). During the storage and sale of chilled meat, including fish products, a significant amount of exudate may be produced due to respiration or the penetration of water vapor (Gaspar et al. 2019); the exudate is rich in various nutrients that promote bacterial growth, negatively impacting the color, flavor, taste, and safety of chilled meat products (Li et al. 2021). Microbial contamination, often resulting from leftover blood and tissue fluids in chilled meat, is the primary factor that contributes to spoilage and can lead to food-borne illnesses (Hultman et al. 2015). One of the key components in modern meat packaging is the absorbent pad, which is designed to absorb these fluids and subsequently maintains the overall integrity of meat products and ensures that the meat is still safe for consumption (Jiao et al. 2023; Castrica et al. 2020). The traditionally used food-absorbent pads are mainly made from biodegradable and non-degradable synthetic superabsorbent polymers (SAPs), which sometimes lack antimicrobial properties and have low absorbency (Hosseini et al. 2025; Jiao et al. 2023). The SAPs consist of polyacrylic acid, polyacrylamide, polyacrylonitrile, and their derivatives (Chen et al. 2022). During use, they may release small amounts of residual monomers or cross-linking agents, which can cause problems in some applications specifically in food (Sonkaya et al. 2021). Moreover, they are not renewable and are not easily biodegradable because of their complex cross-linked structures and high molecular weight upon disposal, which can contribute to environmental pollution problems (Chen et al. 2022). For these reasons, the market today is increasingly demanding sustainable and environmentally friendly packaging solutions. Many manufacturers now offer biodegradable absorbent pads, which not only offer the same advantages as traditional pads but also help mitigate the negative impacts of synthetic materials on the environment (Castrica et al. 2020).

Bacterial cellulose (BC) is a natural biopolymer produced by certain aerobic bacteria. It is characterized by unique properties, including a nanofibril/nanoporous structure, a high degree of crystallinity (84–90%), and high purity, in addition to its biodegradability, biocompatibility, renewability, and distinctive mechanical properties (Gedarawatte et al. 2021; Lin et al. 2020; Dirpan et al. 2019; Afreen and Lokeshappa 2014; Cheng et al. 2009). Owing to these remarkable characteristics, BC is a promising environmentally friendly biomaterial used in the production of biodegradable films for food packaging applications (Cazon and Vazquez 2021a and b). It has been classified as "Generally, Recognized as Safe" (GRS) by the Food and Drug Administration (FDA), which gives it a marketing authorization for the food industry (Azeredo et al. 2019; Shi et al. 2014). In general, BC is used as a stabilizer, thickener, gelling agent, and water-binding additive, and in the manufacturing of dietary fibers in the food industry (Shi et al. 2014; Ullah et al. 2016). It is a nontoxic, hydrophilic material with water absorbency (> 90%) and high permeability to liquids and gases (Nguyen et al. 2008). These characteristics ensure the safety of BC when used as an absorbent pad and allow for the transport of substances through the matrix. The incorporation of antimicrobials in BC membranes can create an active packaging system that prevents spoilage, slows deterioration, helps extend the shelf life of food products, reduces the use of synthetic polymers, and reduces environmental pollution (Dashipour et al. 2014; Ferreira et al. 2016; De Azeredo et al. 2009; Nguyen et al. 2008; Tsai et al. 2018; Sulistyo et al. 2023). On the other hand, essential oils (EOs) are antimicrobials that are used as a release system to inhibit microbial growth in food products to improve their quality and safety (Shaaban et al. 2017; Matan 2012). Many studies have indicated that plant volatile oils contain various secondary metabolites, including flavonoids, alkaloids, phenols, aldehydes, terpenoids, and tannins, which are antimicrobial agents. The infusion of BC with natural oregano essential oil exhibited strong and moderate antibacterial activity against *Cronobacter* strains that can be present in food and cause food-borne diseases and food poisoning (Nagmetova et al. 2020). EOs from citrus species (*Rutaceae* spp.) have antimicrobial activity and are used as sweetening agents (Aldana et al. 2014**)**. A previous study revealed that BC-based edible films containing carboxymethyl cellulose (CMC), 2% lemon, lime, and orange juice have moderate antibacterial activity as natural materials for food packaging (Srikandace et al. 2018). Whereas, the BC film improved by ethanol rosemary extract showed high antibacterial activity against *S. aureus* (Bodea et al. 2022).

Depending on this background, the current study aimed to investigate the ability of bioactive absorbent pads (BAPs) consisting of BC nanofibrils impregnated with tangerine essential oil (TEO) to control emerged microbial pathogens associated with fish fillets. The novelty of the research is represented in the combination of active packaging systems alongside gamma radiation technology. This study hypothesizes that applying this combination, as part of a hurdle technology approach, would be more effective in reducing the bacterial load and achieving the microbial quality and safety of fish products.

## Materials and methods

### Preparation of the fish fillet samples

Fish fillet samples (FFS) were collected from different fish processing markets in Cairo, Egypt, transported to the laboratory in an icebox, and stored at 4 °C until microbiological analysis. Twenty-five grams were taken from each FFS, placed in sterile Stomacher bags, and mixed with 250 ml (mL) of 0.1% peptone water at a suitable speed for 120 s in a 400-mL laboratory blender (Stomacher, IUL Instrument, Spain) to a final dilution of 1:10. The samples were then serially diluted and plated in the appropriate medium according to the method outlined by Andrews and Hammack (2003).

### Isolation of fish fillet-borne pathogens

Different specific media were used for the isolation of pathogenic bacterial strains from FFS, including Starch Ampicillin Agar media; Baird Parker Agar Base (Merck, 1.05406), supplemented with egg yolk tellurite emulsion (Merck, 103,785); and MacConkey agar. One milliliter from each serial dilution was spread onto each medium, the plates were incubated for 24–48 h at 37 °C, and the colonies that appeared were purified, examined under a microscope, and stored in glycerol at − 80 °C for further study.

### Identification of bacterial isolates

Four isolates were selected from distinct colonies (one from each medium) for identification and further experiments. The identification was carried out via VITEK2C (0000139C7BED), Microbiology and Immunology Lab, Biological Prevention Department, Chemical Warfare Main Laboratories, Ministry of Defense, Cairo, Egypt.

### Essential oil extraction

Citrus peels from tangerines, mandarins, and lemons were collected and hydrodistilled for 4 h via a Clevenger-type apparatus to extract the essential oils (Senthilkumar et al. 2009). The extracted oils were then dried with anhydrous sodium sulfate and stored at 4 °C in tightly closed glass vials as a stock for further study.

### Antibacterial screening

#### Agar disc diffusion method

The antibacterial potential of citrus peel essential oils was evaluated in triplicate via the paper disc diffusion method (Hussain et al. 2011; Efstratiou et al. 2012). The bacterial strain suspension (100 μL of 10^6^ CFU/mL) was inoculated on the surface of the plate count agar medium, and filter paper discs (6 mm diameter) loaded with 10 μL of each essential oil were placed on the surface of the solidified medium. The EOs were used as a positive control, while paper discs with sterile water were used as a negative control. The plates were incubated at 37 °C for 24–48 h, and the diameter of the inhibition zones was recorded (mm).

### Evaluation of the minimum inhibitory and bactericidal concentrations

The broth microdilution method (with slight modifications) was used to determine both the MIC and MBC as described by Bouhdid et al. (2008). A volume of 50 μL per well was prepared from serial dilutions of the essential oil stock solution to yield concentrations ranging from 0.024 to 400 μL/mL, which were prepared in sterile 96-well plates. Then, 50 μL of Luria–Bertani (LB) broth medium inoculated with the bacterial suspension adjusted to 10^6^ CFU/mL was added to each well and incubated at 37 °C for 18 h. The bacterial growth of each well was rated by the addition of 5 μL resazurin, and then the mixture was incubated for another 2 h. The MIC was taken as the lowest concentration of essential oil that did not cause any change in resazurin color. Approximately 10 μL from negative wells was subcultured on plate count agar (PCA) medium and incubated for 24 h to carry out the broth dilution tests. The MBC was taken as the lowest concentration of EO with no visible bacterial colonies after 24 h of incubation.

### Production of bacterial cellulose (BC)

Bacterial cellulose (BC) was produced via a symbiotic culture of bacteria and yeast (SCOBY) containing *Acinetobacter lowfi* and *Candida krusei*, respectively, which were isolated from commercial kombucha. It was subsequently grown in Hestrin Schramm (HS) broth medium (Hestrin and Schramm 1954) to prepare the inoculum medium inoculated in 60 mL of sugared water (SW) medium (Hamed et al. 2023) and incubated at 30 °C under static conditions until the BC membrane reached the desired thickness. The BC membrane was harvested, purified using 0.1 N NaOH in an 80 °C water bath for 1 h, neutralized with 4% acetic acid and distilled water, sterilized by autoclaving for 15 min at 120 °C and stored at 4 °C for further study.

### Preparation of antibacterial BC discs

#### Impregnation of BC discs with TEO

To prepare the BC discs for the antibacterial test, some water could be withdrawn from the BC membrane via a clean microfiber towel to permit the absorption and saturation of the BC discs with TEO. To control the amount of withdrawn water and the amount of TEO absorbed, the dimensions of the BC membranes were adjusted (0.5 mm thickness and 6 mm diameter), and the BC membranes were then sterilized by autoclaving for 15 min at 120 °C. For the antimicrobial activity test, the discs (in triplicate) were immersed in 5 mL of different TEO concentrations (400, 800, 1000, and 1600 µL/mL) under the same conditions. Tween 80 was used as an emulsifying agent to facilitate the immobilization of TEO at a 1:1 ratio (TEO:T80). BC discs immersed in sterile distilled water were used as a negative control, while Tween 80 was used without TEO as a control to identify their effect separately, whereas BC discs with pure TEO without emulsifying agents were used as a positive control according to Junka et al. (2019), with some modifications. The discs were immersed for 24 h at 25 °C in a tightly closed container to prevent evaporation of the oil, after which the antimicrobial activity was investigated via the disc diffusion method as previously described.

#### Preparation of bioactive absorbent pads (BC-BAPs)

The BC-BAPs were prepared in the same manner as the BC discs but with different dimensions (5 cm in diameter and 5 mm in thickness). The BC pads were immobilized with different volumes (100, 200, 300, 400, or 500 µL) of emulsified TEO at a concentration of 800 µL/mL (1:1) under aseptic conditions. The absorbance of BC was monitored until it reached saturation without leaving excess TEO on the surface to calculate the desired volume. This method was found to be more efficient for incorporating an equal ratio of oil to saturate BC membranes.

#### Effects of active BC- pads, gamma irradiation and their combination on bacteria artificially inoculated in fish fillets in a model food system

This study used fish fillets as a model food system to investigate the antibacterial properties of active BC pads (BC-BAPs) independently and in combination with gamma irradiation against the isolated bacterial strains (Fig. 5).

#### Preparation of the fish fillet samples

To achieve decontamination of FFS before artificial inoculation, 10 g samples were irradiated at a dose of 10 kGy (5 mA, 8.86 m/min) via an ICT linear electron beam accelerator (3 MeV, energy of 90 kW, beam ampere maximum of 30 mA, maximum conveyor speed of 16 mm/min, maximum high voltage of 3000 kv) at the National Center for Radiation Research and Technology, Cairo, Egypt.

#### Preparation of BC-BAPs

The sterile BC pads were immobilized by TEO at the desired concentrations (MICs) under aseptic conditions. The BC-BAP has two sides: one side is smooth, which represents the bottom of the pad, whereas the other side is rough and is placed facing up, in direct contact with the FFS.

#### Artificial inoculation test

**Fig. 1:**
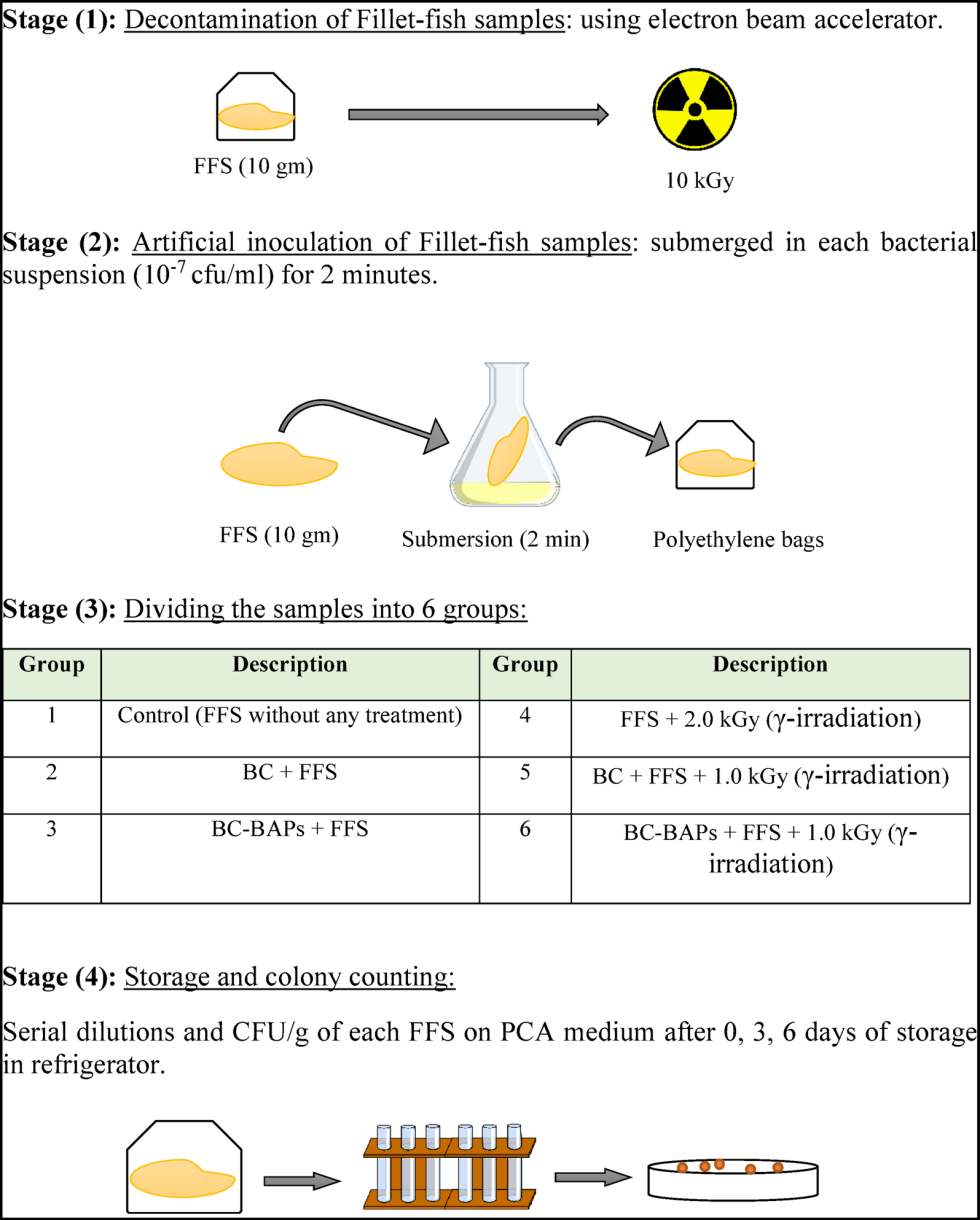
Model food system using fish fillet samples (FFS), bacterial cellulose loaded with tangerine essential oil as bioactive absorbent pads (BC-BAPs) and gamma irradiation

The artificial inoculation was performed by submerging the FFS in nutrient broth containing 10^7^ CFU/mL of each bacterial isolate for 2 min. Afterward, the samples were drained and dried under aseptic conditions within a biosafety cabinet. The inoculated FFS were then divided into six experimental groups for application in a model food system, as summarized in Fig. 1. Gamma irradiation of FFS was conducted via a Cobalt-60 (^60^Co) Cell GC 220 produced by Canada Co. Ltd. and located at the National Center for Radiation and Technology (NCRRT) in Nasr City, Cairo, Egypt.

The samples were exposed to different doses of gamma irradiation (1.0 and 2.0 kGy) at ambient temperature, where the dose rate was 0.644 kGy/h. After treatment, the samples were placed in polyethylene bags and stored in a refrigerator. Sampling was carried out on days 0, 3, and 6 of storage to determine the inoculated bacterial count on the PCA medium according to the SR ISO 4833/2014 standard. The SR EN ISO 7218/2014 standard was used for microbiological examinations and interpretation of the results.

### Characterization of irradiated and non-irradiated TEO and BC-BAPs

#### Determination of the chemical constituents of TEO via GC‒MS analysis

The TEO sample was exposed to gamma irradiation at a dose of 1.0 kGy, where the dose rate was 0.555 kGy/h. The irradiated and non-irradiated samples were analyzed via the GC‒MS technique in the Central Lab of Chromatography-Radioisotope Application Division-Nuclear Research Center (EAEA) via an Agilent Technology 8890GC equipped with an injector autosampler coupled to an Agilent 5977B series MSD. GC column 122–5562: DB-5 ms, 60 m × 250 µm × 0.25 µm. The chromatograms were analyzed via the MSD MassHunter software package, and the mass spectra were compared and matched with the National Institute of Standards and Technology (NIST) Library (NIST20).

#### Fourier transform infrared (FTIR) spectroscopy of BC-BAPs-immobilized TEO

BC-BAPs were analyzed in FTIR-KBr mode via a BRUKER VERTEX 70 device allocated to the Central Labs National Center for Radiation Research and Technology (NCRRT), Egyptian Atomic Energy Agency (EAEA), Cairo, Egypt. Structural identification of the irradiated and non-irradiated samples was carried out, and the results were compared with those of the control sample. The spectral profiles of all the dried samples were carried out at room temperature in the range of 4000–400 cm^−1^.

### Statistical analysis

Data were analyzed using the SAS software package, version 9 (SAS 2002). Statistical significance between treatment groups was assessed via analysis of variance (ANOVA), with post hoc comparisons performed by Duncan’s multiple range test (Duncan 1955). A significance level of P ≤ 0.05 was considered statistically significant. All experiments were conducted with three independent replicates, and the results are presented as the means ± SDs (n = 3) to ensure robust data collection.

## Results

### Isolation and identification of selected bacterial strains from FFS

The isolation and identification of bacteria from fish fillet samples using VITEK 2C revealed the presence of unusual foodborne pathogens. The results in Table 1 revealed that 7 samples were positive for *K. oxytoca*, with numbers as high as 10^5^ CFU/g, whereas* K. rosea* appeared in 4 samples, with counts not exceeding 10^2^ CFU/g. Five samples were positive for *S. ficaria* at concentrations ranging around 10^2^–10^4^ CFU/g, and 8 samples contained the *E. cloacae* complex, with counts ranging from 10^2^–10^3^ CFU/g.

**Table 1:** Number of bacterial pathogenic strains isolated from the fish fillet samples (CFU/g)

FF samples	Bacterial population in CFU/g (Means ± SDs)			
	*K. oxytoca*	*S. ficaria*	*E. cloacae*	*K. rosea*
1	5.31 ± 0.44	4.31 ± 0.44	2.39 ± 0.07	ND
2	5.30 ± 0.42	4.31 ± 0.44	3.53 ± 0.03	ND
3	2.38 ± 0.12	ND	2.97 ± 0.03	ND
4	3.31 ± 0.44	2.39 ± 0.03	ND	ND
5	2.30 ± 0.21	ND	ND	2.50 ± 0.22
6	ND	ND	2.48 ± 0.69	2.15 ± 0.21
7	4.31 ± 0.31	ND	2.15 ± 0.21	1.64 ± 0.13
8	4.40 ± 0.12	2.30 ± 0.42	3.82 ± 0.24	1.50 ± 0.28
9	ND	3.38 ± 0.12	3.37 ± 0.05	ND
10	ND	ND	3.33 ± 0.47	ND
Frequency %	70	50	80	40

*ND* not detected.

### Antibacterial activity of citrus peel EOs against isolated strains

The current investigation examined the antibacterial activity of essential oils extracted from tangerine, mandarin, and lemon peels (TEO, MEO, and LEO, respectively) against previously isolated bacteria (*K. oxytoca, S. ficaria E. cloacae,* and* K. rosea*) via a disk diffusion assay. All the tested citrus EOs demonstrated activity against isolated bacteria, with inhibition zones ranging from 9.1 to 17.1 mm.

The data in Table 2 show significant differences in the inhibition zones of citrus peel EOs against the tested bacteria, where TEO and LEO EOs showed higher antibacterial efficacy than MEO. However, *E. cloacae* exhibited the lowest sensitivity to TEO, LEO, and MEO, with inhibition zones of 10.4, 10.2, and 9.3 mm, respectively. In contrast, *K. rosea* was the most sensitive of the tested isolates, as evidenced by the largest inhibition zones of 17.1, 16.2, and 11.3 mm for TEO, LEO, and MEO, respectively.

**Table 2:** Antibacterial activity of essential oils extracted from tested citrus peels against pathogenic strains isolated from fish fillet samples

Inhibition zone (mm)				
Bacterial strains	*K. oxytoca*	*S. ficaria*	*E. cloacae*	*K. rosea*
EOs				
TEO	13.4^c^_a_ ± 0.11	14.7^d^_a_ ± 0.13	10.4^a^_a_ ± 0.11	17.1^b^_a_ ± 0.42
MEO	12.1^c^_b_ ± 0.31	9.1^d^_b_ ± 0.12	9.3^a^_b_ ± 0.13	11.3^b^_b_ ± 0.21
LEO	12.2^ cc^ ± 0.21	13.2^d^_c_ ± 0.11	10.2^a^_c_ ± 0.31	16.2^b^_c_ ± 0.12

Diameter of the disc = 6 mm

The values are the means of the inhibition zones in triplicate ± standard deviations (SDs). Different letters indicate significantly different values (*p* < 0.05), where the superscripts indicate the difference within rows (the various strains with the same essential oil), and the subscripts indicate the difference within columns (the various essential oils with the same strain).

Overall, the results confirmed that the tested citrus peel EOs had a greater effect on the gram-positive bacteria than on the gram-negative bacteria of the isolates under investigation. In this context**,** TEO was selected for additional tests because of its effectiveness against isolated bacteria, the large quantity of peel waste from which oil can be extracted, and the lack of research on this topic.

### Determination of the MIC and MBC of TEO against the isolated pathogens

**Fig. 2:**
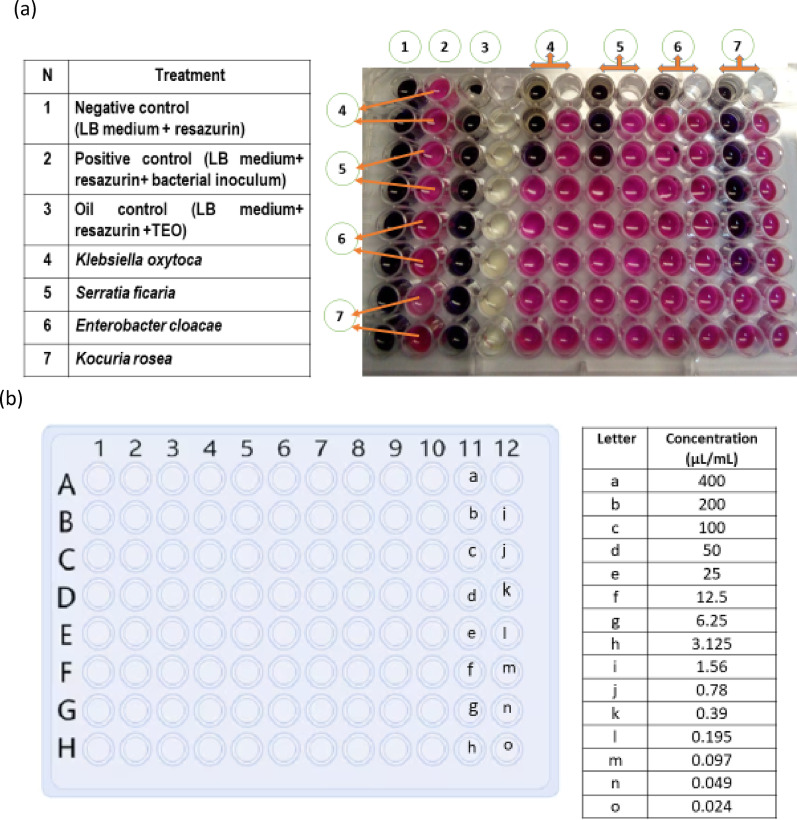
MIC of TEO against pathogenic strains isolated from fish fillet samples via the broth microdilution method (**a**); the concentration order is indicated in detail (**b**)

The MIC and MBC values of TEO against previously isolated bacteria are presented in Table 3 and Fig. 2. The MICs resulting in complete growth inhibition of *E. cloacae, K. oxytoca,* and *S. ficaria* were 400, 100, and 100 µL/mL, respectively, whereas for *K. rosea,* the MIC was 12.5 µL/mL. In general, the results revealed that the MBC in this study was equal to or greater than the MIC. The MBC of *E. cloacae* was 400 µL/mL (the same as the MIC value), whereas it was higher than the MIC values for *K. rosea*, *K. oxytoca,* and *S. ficaria* (200, 400, and 200 µL/mL, respectively). Additionally, the MIC of TEO was more effective against gram-positive bacteria than against gram-negative bacteria.

**Table 3:** MICs and MBCs of tangerine essential oil against pathogenic strains isolated from fish fillet samples (µL/mL)

MIC and MBC of TEO	Bacterial strains			
	*K. oxytoca*	*S. ficaria*	*E. cloacae*	*K. rosea*
MIC (µL/mL)	100	100	400	12.5
MBC (µL/mL)	400	200	400	200

### Production of BC and preparation of BC discs and pads

**Fig. 3:**
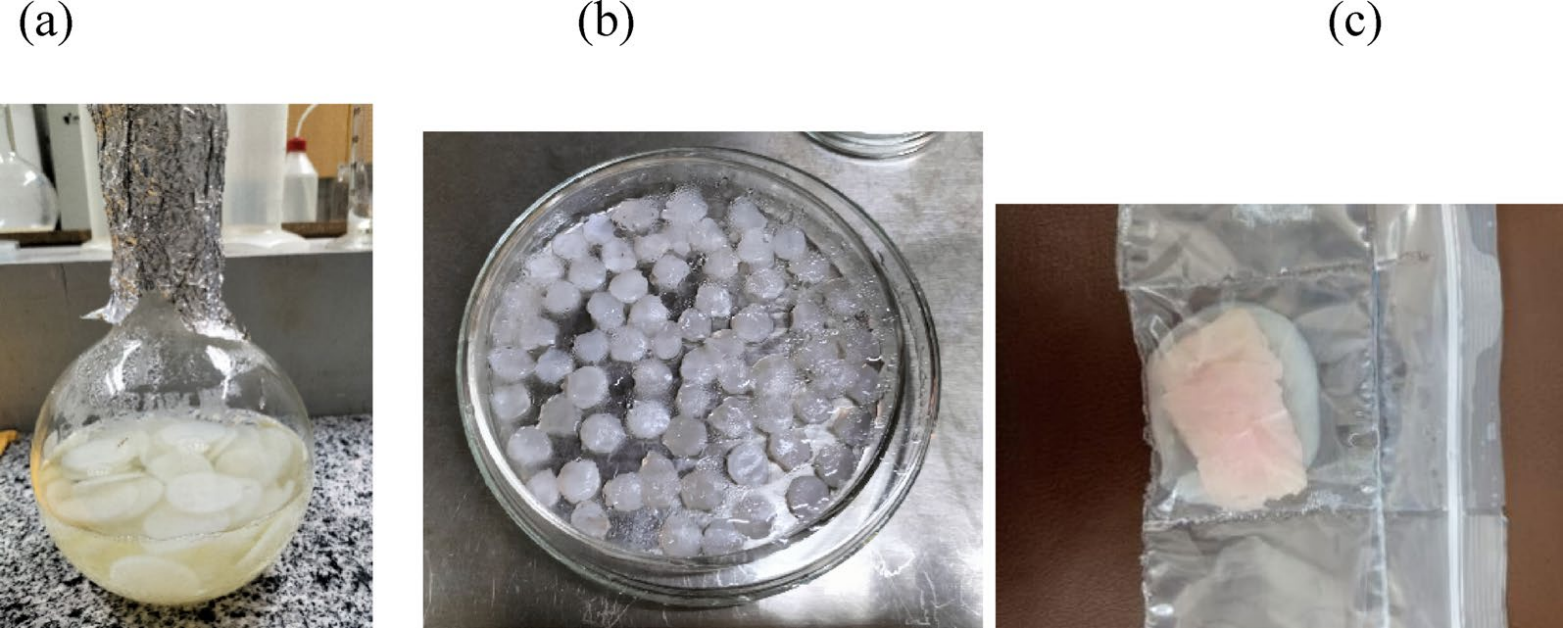
The biosynthesis of the BC membrane in sugared water medium (**a**), preparation of BC discs for antimicrobial tests (**b**), and BC pads for application tests (**c**)

Bacterial cellulose (BC) formed on the surface of the air‒medium interface after incubation for 21 days, reaching a thickness of 5.0 mm and a diameter of 5.0 cm. After harvesting and purification, the BC appeared as a white hydrated cohesive membrane, and the BC discs and pads were prepared as described in the methodology and illustrated in Fig. 3.

### Antibacterial activity of BC discs impregnated with TEO

The data in Table 4 revealed that the BC loaded with emulsified TEO had more efficient antibacterial activity than the non-emulsified BC. Moreover, the concentration of 800 µl/mL had the optimum inhibition zone for all the isolated strains, ranging between 12.7 ± 0.58 and 14.3 ± 2.08 mm. On the other hand, no inhibition zone was detected for BC immobilized by pure TEO. The data (Table 4) also indicate that the clear zone area of the BC/TEO discs (12.5 mm) decreased compared with the clear zone of the filter paper discs containing TEO (17 mm) for *K. rosea*. There was an increase in the inhibition zone size for *E. cloacae*, increasing from approximately 10 mm to 14 mm when TEO was applied to filter paper discs and BC/TEO, respectively.

**Table 4:** Antimicrobial activity of BC discs impregnated with different concentrations of TEO (µL/mL) against pathogenic strains isolated from fish fillet samples

Treatments of BC discs	Bacterial strains			
	*K. oxytoca*	*S. ficaria*	*E. cloacae*	*K. rosea*
Dist. Water	–	–	–	–
Tween 80 (T80)	–	–	–	–
TEO	–	–	–	–
EO/H_2_O (400 µL/mL)	–	–	–	–
EO/H_2_O (800 µL/mL)	–	–	–	–
EO/H_2_O (1000 µL/mL)	–	–	–	–
EO/T80 (400 µL/mL)	–	–	13.3^a^_a_ ± 2.08	–
EO/T80 (800 µL/mL)	13.3^c^_a_ ± 0.57	13.0^d^_a_ ± 1.00	14.3^a^_b_ ± 2.08	12.7^b^_a_ ± 0.58
EO/T80 (1000 µL/mL)	–	12.3^c^_b_ ± 0.57	13.6^a^_c_ ± 1.52	12.6^b^_b_ ± 0.57

DW: Distilled water T80: Tween 80 TEO: Tangerine essential oil

The values are the means of the inhibition zones (in triplicate) ± standard deviations (SDs). Different letters indicate significantly different values (*p* < 0.05), where the superscripts indicate the difference within rows (the various strains with the same treatment) and the subscripts indicate the difference within columns (the various treatments with the same strain)

To create bioactive absorbent pads (BAPs), the bacterial cellulose (BC) membrane was immersed in emulsified TEO. Observations indicated that the saturation of partially dehydrated BC pads with 200 µL of emulsified TEO was optimal, as this volume effectively saturated the BC without leaving any excessive oil.

### Effects of active BC-pads and gamma irradiation and their combination on pathogenic isolates of artificially inoculated fish fillets in a model food system

The data in Table 5 show the inhibitory effects of BC/TEO pads, gamma irradiation and their combination on the initial population counts of four isolated bacterial strains that were artificially inoculated into the fish fillet samples and stored at 4 ± 1 °C for 6 days. On day one, i.e., after the inoculated fish were placed in the bags for 2 h, the initial population counts of the control samples (samples without any treatment) were 5.47, 5.63, 5.14, and 5.25 logs CFU/g for *K. oxytoca*, *S. ficaria, E. cloacae,* and* K. rosea*, respectively. The microbial loads of the fish fillet samples on the BC pad were 5.43, 5.04, 5.07, and 5.07 log CFU/g, respectively, which were lower than those of the control samples.

**Table 5:** Effects of active BC-pads and gamma irradiation individually and their combination on the log bacterial count in fish fillet samples as a model food system

Treatments Days	Control	BC	BC + TEO	2 kGy	BC + 1.0 kGy	BC + TEO + 1.0 kGy
*K. oxytoca*						
0	5.47^a^_a_ ± 0.01	5.43^b^_a_ ± 0.02	4.49^c^_a_ ± 0.01	2.23^d^_a_ ± 0.04	3.74^e^_a_ ± 0.03	2.32^f^_a_ ± 0.02
3	7.17^a^_b_ ± 0.05	6.81^b^_b_ ± 0.04	5.07^c^_b_ ± 0.02	3.25^d^_b_ ± 0.02	4.56^e^_b_ ± 0.02	3.30^f^_b_ ± 0.01
6	8.89^a^_c_ ± 0.02	7.30^b^_c_ ± 0.03	5.68^ cc^ ± 0.02	4.69^d^_c_ ± 0.01	5.36^e^_c_ ± 0.04	3.92^f^_c_ ± 0.04
*S. ficaria*						
0	5.63^a^_a_ ± 0.02	5.04^b^_a_ ± 0.03	4.47^c^_a_ ± 0.01	2.47^d^_a_ ± 0.05	3.81^e^_a_ ± 0.03	2.04^f^_a_ ± 0.01
3	7.72^a^_b_ ± 0.04	6.00^b^_b_ ± 0.02	5.02^c^_b_ ± 0.02	3.55^d^_b_ ± 0.01	4.77^e^_b_ ± 0.02	3.38^f^_b_ ± 0.02
6	8.91^a^_c_ ± 0.01	7.23^b^_c_ ± 0.04	5.79^ cc^ ± 0.04	4.00^d^_c_ ± 0.02	5.65^e^_c_ ± 0.02	3.89^f^_c_ ± 0.03
*E. cloacae*						
0	5.14^a^_a_ ± 0.01	5.07^b^_a_ ± 0.02	4.07^c^_a_ ± 0.01	2.82^d^_a_ ± 0.02	3.47^e^_a_ ± 0.04	2.17^f^_a_ ± 0.01
3	6.20^a^_b_ ± 0.04	6.91^b^_b_ ± 0.02	4.82^c^_b_ ± 0.03	3.84^d^_b_ ± 0.03	4.39^e^_b_ ± 0.01	2.96^f^_b_ ± 0.02
6	8.72^a^_c_ ± 0.02	6.80^b^_c_ ± 0.01	5.36^ cc^ ± 0.01	4.60^d^_c_ ± 0.02	5.46^e^_c_ ± 0.02	3.77^f^_c_ ± 0.02
*K. rosea*						
0	5.25^a^_a_ ± 0.02	5.07^b^_a_ ± 0.03	4.36^c^_a_ ± 0.04	2.46^d^_a_ ± 0.01	3.77^e^_a_ ± 0.01	2.89^f^_a_ ± 0.02
3	6.73^a^_b_ ± 0.01	5.95^b^_b_ ± 0.04	4.50^c^_b_ ± 0.01	2.90^d^_b_ ± 0.02	4.20^e^_b_ ± 0.03	3.49^f^_b_ ± 0.04
6	8.69^a^_c_ ± 0.02	6.34^b^_c_ ± 0.01	4.97^ cc^ ± 0.02	3.93^d^_c_ ± 0.04	5.32^e^_c_ ± 0.02	3.86^f^_c_ ± 0.05

The values are the means of the log bacterial counts in triplicate ± standard deviations (SDs). Different letters indicate significantly different values (*p* < 0.05), where the superscripts indicate the difference within rows (the various treatments with the same incubation period) and the subscripts indicate the difference within columns (the various incubation periods with the same treatment) for each strain individually

After six days of storage, compared with the control, the BC pads reduced the initial load of the four bacterial strains by 1.59, 1.68, 1.92, and 2.35 log CFU/g, respectively. However, the BC/TEO bioactive pads reduced the bacterial counts of the artificially inoculated strains after loading for 2 h (on day one) by 0.98, 1.16, 1.07, and 0.89 log CFU/g, respectively. On the other hand, the BC/TEO pads reduced the initial load of the four bacterial isolates by 3.21, 3.12, 3.36, and 3.72 log CFU/g, respectively, after six days of storage. In contrast, the growth curve of the bacteria generally tended to increase over storage, with the counts in the control samples reaching 8.89, 8.91, 8.72, and 8.69 log CFU/g, respectively, on day six. On the basis of these findings, the BC/TEO composite pad had a major effect on the bacterial count. As demonstrated in Table 5, gamma irradiation at 2.0 kGy reduced the initial load of *K. oxytoca, S. ficaria, E. cloacae,* and* K. rosea* in inoculated fish fillet samples by 3.24, 3.16, 2.32, and 2.79 log cycles, respectively, compared with the control. However, after six days of storage, the initial loads of the four bacterial isolates were reduced by 4.20, 4.91, 4.12, and 4.76 log cycles, respectively. The results demonstrated the effectiveness of gamma irradiation as a preservation method, which significantly inhibited the growth of the tested bacteria. The effects of the combination of gamma irradiation and BC/TEO pads on the growth of the four bacteria mentioned above in the fish fillet samples are also displayed in Table 5. After the inoculated samples were placed onto the BC and BC/TEO pads, they were irradiated at 1.0 kGy. On the first day, the surviving populations of the four bacterial isolates decreased because of the combined treatments. These populations are estimated to be 3.74, 3.81, 3.47, and 3.77 log cycles (BC + 1.0 kGy) and 2.32, 2.04, 2.17 and 2.89 log cycles (BC/TEO + 1.0 kGy) of *K. oxytoca, S. ficaria, E. cloacae,* and *K. rosea,* respectively. However, after 6 days, the population counts of the tested bacteria decreased by 3.53, 3.26, 3.26, and 3.37 log cycles for BC + 1.0 kGy and 4.97, 5.02, 4.95, and 4.83 log cycles for BC/TEO + 1.0 kGy, respectively. According to these results, the combination treatment had a significant (*p* < 0.05) synergistic effect with TEO as an antimicrobial agent on the population counts of all tested bacteria after six days of storage compared with the control.

### GC/MS analysis of tangerine essential oil (TEO)

**Fig. 4:**
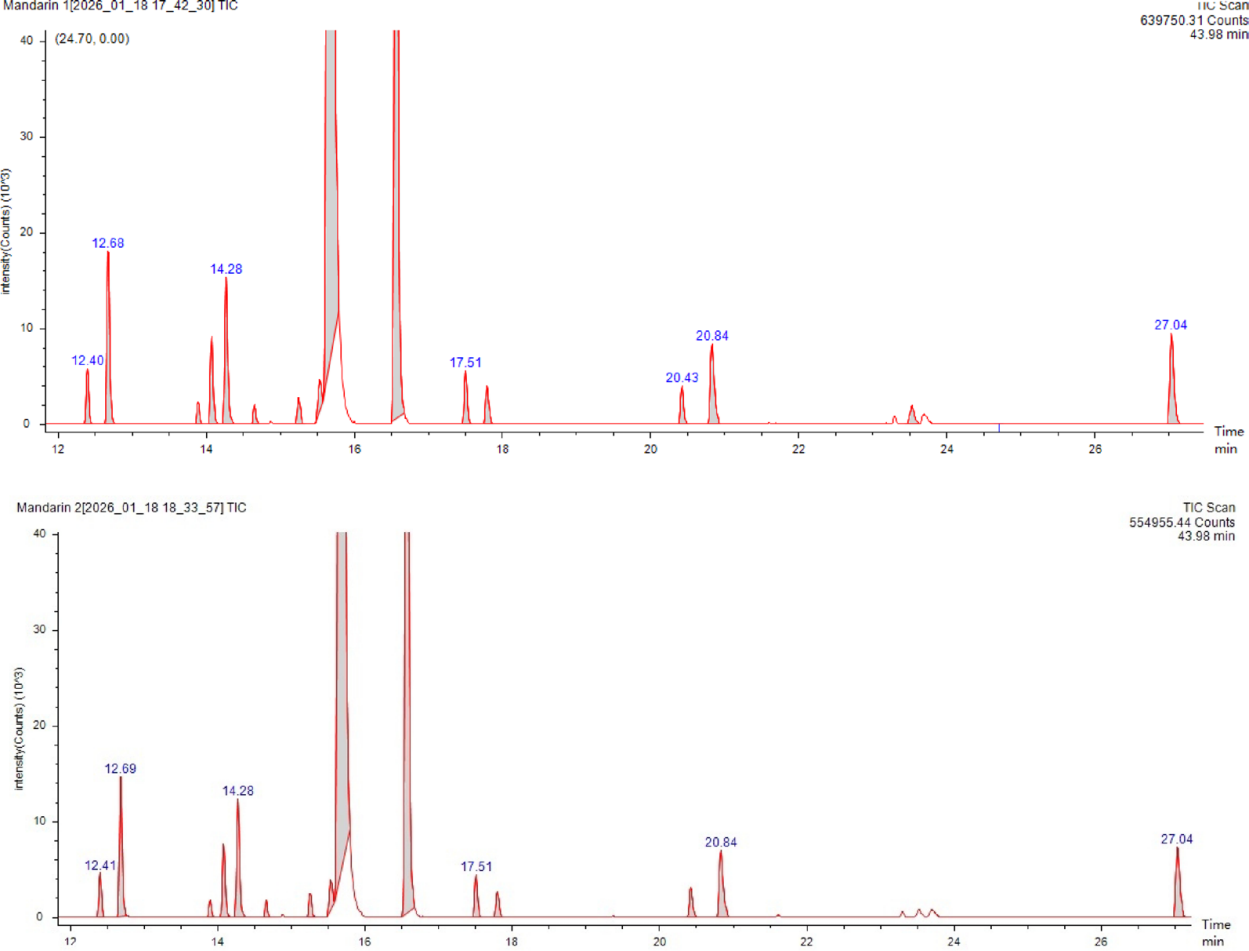
GC‒MS chromatograms of irradiated and non-irradiated TEO

The analysis of TEO via GC/MS revealed the presence of 16 different chemical constituents, with the monoterpene hydrocarbon group representing the main constituents (Table 6 and Fig. 4). Two major peaks were observed at 15.7 and 16.6 min, corresponding to 79.9% D-limonene and 11.9% γ-terpinene, respectively. The lowest peak was attributed to octanal (at 14.7 min at 0.1%). No significant changes between the samples subjected to gamma irradiation at 1.0 kGy and non-irradiated samples were observed, except for the appearance of peak 15 (3H-indazol-3-one, 1, 2, or dihydro-1-methyl) at 23.5 min with 0.2% after irradiation.

**Table 6:** Chemical composition of irradiated and non-irradiated TEO via GC‒MS analysis

Peak index	Retention time	Compound name	Tangerine (1.0 kGy)	Tangerine (control)
			(%)	(%)
1	12.4	α-Thujene	0.43	0.42
2	12.68	α-Pinene	1.43	1.42
3	13.9	ß-Phellandrene	0.17	0.15
4	14.08	ß-Pinene	0.74	0.77
5	14.28	ß-Myrcene	1.31	1.27
6	14.66	Octanal	0.14	0.14
7	15.25	(+)-4-Carene	0.24	0.25
8	15.55	ρ-Cymene	0.31	0.35
9	15.72	D-Limonene	79.94	80.00
10	16.58	γ-Terpinene	11.87	12.17
11	17.51	Isoterpinolene	0.46	0.45
12	17.8	Linalool	0.36	0.29
13	20.43	Terpinen-4-ol	0.35	0.34
14	20.83	L-α-Terpineol	0.95	0.96
15	23.53	3H-Indazol-3-one, 1,2-dihydro-1-methyl-	0.24	0.00
16	27.04	Methyl methanthranilate	1.06	1.02
	Total	16	100.00	100.00

#### FTIR analysis of BC and BC/TEO

The FTIR spectrum of BC in Table 7 and Fig. 7 (SM1), revealed no changes between the BC samples before and after irradiation at a dose of 1.0 kGy, whereas some changes were recorded after impregnation of BC with TEO, especially in the –OH region (3168–3470 cm^−1^ and 3351–3558 cm^−1^ for the non-irradiated and irradiated BC/TEO samples, respectively).

**Table 7:** Correlation between FTIR spectra and GC/MS analysis results for irradiated and non-irradiated BC and BC/TEO samples

Wavelength cm^−1^				Functional group	Interpretation
BC	BC*	BC/TEO	(BC/TEO)*		
3290.9	3214.6	3168.7	3351.9	–OH	Hydroxyl group stretching vibration of the main BC structure
3335.9		3213.6	3396.8		
		3267.7			
		3425.8 3470.9	3513.9		Bending of alcoholic compounds (linalool, terpinen-4-ol, α-terpineol, Octanal)
–	–		3558.8		
2901.3	2900.7	2871.5	2941.9	C–H	Stretching of alkane and asymmetric CH_2_ stretching of BC
		2826.2			Asymmetric stretching vibration of the C–H bond in the methyl (–CH_3_) and methylene (–CH_2_) groups in limonene and terpinene (TEO)
					–CH₂ (acyl chain of fatty acid) of Tween 80
2721.7	2721.8	–	–CH_2_	Symmetric stretching of BC structure	
–	–	1734	1732	–C = O	Aldehyde/ester groups or carbonyl group deformation vibration
					Ester moiety of Tween 80
1572.6	1572.3	1588.7	1586.6	–OH	Deformation of BC
					Vibrations of aromatic nuclei from TEO
1422.6	1422.9	–	1458	–CH_2_	Deformation of BC structure
				C–H	Peaks approximately 1450 cm^−1^, represent the doublet stretching of the C–H bond in the methylene groups aromatic compounds (limonene and terpinene)
	1365.7	1331	–	–CH_3_	Deformation of BC structure
					Methylene bending of TEO that disappeared after irradiation which indicates its cleavage
	1244	1286	–	–CH	Deformation of BC
					TEO–Radiation effect
1160.4	1160.9	–	–	C–O–C	C–O–C asymmetric and C–O stretching at β-glycosidic linkage
				C–O	Disappeared after BC/TEO indicating their cleavage and potential reaction with oil
–	–	–	945	C–H	Bending vibration key absorbtion band of γ-terpinene compound
895	895.2	850.9	850	β (1–4)	Glycosidic bond (vibration) of BC structure
				= C–H	
					Terminal methylene of aromatic limonene and terpinene compounds (out of plane bending)

There are two peaks in the region approximately 2900 cm^−1^ in both the non-irradiated and irradiated BC samples, while it was recognized at 2941 cm^−1^ in the irradiated BC/TEO samples and appeared as 2 peaks at 2826 and 2871 cm^−1^ in the BC/TEO samples. Additionally, absorption peaks approximately 2721.7 cm^−1^ were observed in both the non-irradiated and irradiated BC samples, whereas they disappeared in both the BC/TEO samples.

Moreover, some distinct peaks appeared in the ranges of 1572–1586 cm^−1^, 1422–1458 cm^−1^ and 1365.7 cm^−1^. There are intense peaks at 1734–1732 cm^−1^ in both the non-irradiated and irradiated BC/TEO samples. On the other hand, two absorbance peaks at wavelengths of 1588.7 and 1586.6 cm^−1^ appeared in the presence of TEO. However, a peak in the region of 945 cm^−1^ appeared in the irradiated and non-irradiated BC/TEO samples (i.e., after the addition of TEO).

## Discussion

The future of absorbent pads as a part of food packaging is evolving rapidly. It combines innovation, efficiency, and eco-friendly design that is based on technology and sustainability. The current study investigated the application of BC-impregnated TEO as a bioactive absorbent pad for reducing/inhibiting the growth of emerged pathogenic bacterial strains contaminating fish fillets. Table 1 shows that 24 pathogenic bacteria were isolated from 10 samples of FFS, of which 8 isolates were identified as *Enterobacter cloacae* complexes, 7 isolates were *Klebsiella oxytoca,* 5 isolates belonged to *Serratia ficaria,* and 4 isolates were *Kocuria rosea*. In contrast to earlier studies, variations in the types and numbers of bacteria in the FFS were noted (Ghanem et al. 2019; Da Silva et al. 2020; Mitiku et al. 2022). In recent years, microbial diversity has changed, and new antibiotic-resistant pathogenic bacteria have emerged in animals, including marine fish, which is a major health problem worldwide. These changes are attributed to human activity, climate change, and environmental problems such as pollution and reverse the cross-transmission of pathogens between animals and humans (Bukha et al. 2025). For example, information on *K. oxytoca* in aquatic products was uncommon until the years 2018–2019, when it was first studied in aquatic and benthic organisms (Ni et al. 2021), but it has recently become a major concern as the cause of many fish-borne infections (Cortés-Sánchez et al. 2025). In addition, multidrug-resistant *K. oxytoca* and *E. cloacae* have been isolated from fish samples collected from the southeast coast of India (Kamala and Sivaperumal 2023). Another study isolated *E. cloacae* and *K. oxytoca* from raw Nile fish collected from different markets in Benha, Egypt, which corroborated the results of our study (Rawash et al. 2019). Similarly, in Zambia, antibiotic-resistant *Enterobacter* and *Serratia* species have been isolated from fresh fish fillets (Mwendelema and Hang'ombe 2025). None of the common pathogenic bacteria, including *Salmonella, L. monocytogenes*, *Vibrio* spp., and *E. coli,* were detected in the strains isolated from farmed shrimp in Bangladesh. However, *Enterobacter cloacae*, *Escherichia fergusonii, Proteus penneri, Klebsiella aerogenes, Enterococcus faecalis, Serratia marcescens, Citrobacter freundii*, and *Aeromonas dhakensis* are commonly identified (Khan et al. 2024).

The multidrug-resistant *E. cloacae* has recently been isolated from fish, particularly mullets (*Mugil* spp.), as an emerging pathogen (Mabrok et al. 2024). *E. cloacae* is a gram-negative bacterium that causes severe disease and death in various fish species; it is resistant to several antibiotics, including ampicillin, and has been isolated from fish tissues as an indicator of water contamination (Brouwer et al. 2018; Topić Popović et al. 2019). *K. rosea*, a gram-positive bacterium, has been recognized as a foodborne pathogen that is able to adapt its metabolic activities, such as high levels of ionizing radiation or toxic metals, to survive in harsh environments and can persist throughout the food chain from farms to consumers (Timkina et al. 2022; Detcharoen et al. 2025). A high microbial load in food products can generally be caused by poor personal hygiene during harvesting, processing, and filleting due to inadequate handling during transportation (Penakalapati et al. 2017; FDA 2021; Luqman et al. 2024). According to the Centre for Food Safety (CFS 2014), microbial loads of less than 20 colony-forming units (CFUs) per g are considered satisfactory, 20 to 10^2^ CFU/g are intermediate or borderline, and anything above 10^2^ CFU/g is considered unacceptable for human consumption. Therefore, this study revealed that the average microbial load of the fish fillet samples was within the unacceptable range. The number of pathogenic bacteria in the FFS exceeded both Egyptian and international microbiological standards (EOSQC/8891/2005; FAO, 2020). Previous studies have shown that the microbial contamination rate is higher in ready-to-eat products than in whole fish, probably as a result of the cutting and preparation process (Lerma-Fierro et al. 2020; Mitiku et al. 2022; Prado-Toledo et al. 2022; Luqman et al. 2024). For example, a study by Hafez and colleagues revealed that the prevalence of *A. hydrophila* is significantly greater in fish fillets (33.3%) than in whole fish (10.5–12.5%) (Hafez et al. 2018). The prevalence of microbiological spoilage presents a serious issue, as it is a primary cause of food waste, foodborne diseases, and even death. Thus, finding new and effective ways to stop and remove this contamination is vital. Moreover, the increasing emphasis on social responsibility has led consumers to prefer natural food items and avoid harmful additives (Hassoun et al. 2024). Several studies have demonstrated the efficacy of essential oils (EOs) as safer multitarget alternatives to synthetic additives and preservatives in various food products owing to their antimicrobial properties, especially with respect to cell membrane damage and lipid peroxidation inhibition (Afidah et al. 2025).

### Antibacterial activity of citrus peel essential oils against isolated pathogens using the broth microdilution method

Egypt is considered one of the largest producers and exporters of citrus fruits in the world. During the production and processing of fresh citrus, a significant amount of waste (peels, seeds, and pulp) is generated, accounting for 50–65% of the fruit (Magalhaes et al. 2023; Vilas-Boas et al. 2022). These fruit processing byproducts (FBPs) are rich in valuable bioactive compounds, which provide significant antioxidant and antimicrobial activity that inhibit food spoilage and foodborne pathogens (Hasan et al. 2024). Citrus peels contain bioactive compounds such as pigments (carotenoids), essential oils (EOs), and polyphenols (Wedamulla et al. 2022).

In this study, the antibacterial activity of essential oils extracted from tangerine, mandarin, and lemon peels (TEO, MEO, and LEO) was examined using the disc diffusion method. The results demonstrated that all the tested citrus essential oils (CEOs) exhibited activity against the isolated bacteria, with inhibition zones ranging from 9 to 17 mm (Table 2). This result is in agreement with previous studies and confirms the efficacy of CEOs against food spoilage (Olatunya et al. 2024; Ibrahim et al. 2024; Sabry et al. 2024). Other research on CEO has shown differing levels of antibacterial effectiveness due to the plant species, cultivation region, time of harvesting, and how the oil was extracted (Değirmenci and Erkurt 2020; Bhandari et al. 2024). Plant volatile oils are composed of diverse secondary metabolites, such as flavonoids, alkaloids, terpenoids, and tannins, which are associated with their antimicrobial effects (Anwar et al. 2023; Ibrahim et al. 2024). These substances function by disrupting protein, nucleic acid, and cell wall synthesis, as well as damaging cell membrane integrity, thereby increasing permeability and ultimately causing cell death through ions and molecules (Kholaf et al. 2017; Simons et al. 2020; Bhandari et al. 2024; Visakh et al. 2022; Ibrahim et al. 2024). The present data revealed considerable differences in how TEO, MEO, and LEO inhibited the tested bacteria, likely due to variations in their chemical compositions and concentrations. These results are consistent with previous research highlighting the varying levels of flavonoids and phenolic compounds in citrus peel extracts, as well as the synergistic effects of bioactive compounds such as limonene, γ-terpinene, α-pinene, linalool, β-pinene, and β-myrcene, which are known for their potential antibacterial properties (Okwu et al. 2007; Ali et al. 2024; Ibrahim et al. 2024; Sabry et al. 2024). These findings indicate that citrus peel essential oils (EOs) are more effective against gram-positive bacteria than against gram-negative bacteria. *K. rosea* exhibited the greatest sensitivity to TEO, LEO, and MEO, whereas *E. cloacae* exhibited the lowest sensitivity. This result aligns with prior research, which attributed the greater resistance of gram-negative bacteria to their complex structure, characterized by higher levels of lipopolysaccharide and phospholipids, than gram-positive bacteria (Ibrahim et al. 2024; Sabry et al. 2024). TEO was selected for subsequent experiments because of its efficacy against the identified pathogenic bacteria, the availability of peel waste for oil extraction, and the limited existing research in this specific area.

### Antibacterial activity of TEO

To evaluate the antimicrobial characteristics of TEO against previously isolated bacteria (*E. cloacae*, *K. rosea*, *K. oxytoca*, and *S. ficaria*), three steps were performed:

(i) Pure TEO was used directly to determine the minimum inhibitory concentration (MIC) and minimum bactericidal concentration (MBC) via the broth microdilution method. (ii) BC discs were tested after being immersed in the MIC of both emulsified and non-emulsified TEO to verify its efficacy when fixed on the discs. (iii) BC nanofibril membranes impregnated with a specific volume and concentration of emulsified TEO were employed as bioactive pads in a model food system (FMS) application experiment.

- MIC and MBC of TEO against isolated pathogenic bacteria

Table 3 illustrates how TEO affects the MIC and MBC of various bacterial strains, where the MBC values were either equal to or greater than the MIC values. For example, the MBC values for *K. rosea*, *K. oxytoca*, and *S. ficaria* were 200, 400, and 200 µL/mL, respectively (exceeding their corresponding MIC values). Conversely, the MBC value for *E. cloacae* was 400 µl/mL (matching its MIC value). Earlier studies have shown that tangerine peel contains high concentrations of essential oils, which are abundant in secondary metabolites such as 0.3131% terpinene, 0.3% linalool, 1.4% pinene, 1.81% sabinene, 3.4% myrcene, and 91.79% limonene, making it highly effective against bacteria (Calo et al. 2014; Farouk et al. 2022; Ali et al. 2024; Olatunya et al. 2024; Sabry et al. 2024). D-limonene is the main compound exhibiting broad-spectrum antibacterial properties in Nigerian tangerine peel, and is effective against both gram-negative and gram-positive bacteria, where the minimum inhibitory concentrations (MICs) of the essential oils vary from 0.025 to 0.15 mg/mL (Ayoola et al. 2008). According to the results of the GC/MS analysis, TEO contains a high percentage (79.9%) of D-limonene and (11.9%) γ-terpinene.

(b) Antibacterial activity of TEO immobilized on BC discs against isolated pathogenic bacteria

Bacterial cellulose is known for its unique features, particularly its porous structure and high permeability to liquids and gases, alongside its capacity to absorb water (Nguyen et al. 2008). It is produced by bacterial cells as nanofibrils (100 nm in diameter and 100 µm long) with ribbon-like structures that self-assemble into a three-dimensional, sponge-like network (Taboas et al. 2003; Czaja et al. 2006; Ul-Islam et al. 2011). In the present study, these properties might help with incorporating, maintaining, moving, and releasing TEO within the BC matrix, as well as absorbing excess fluids from FFS. It is logical that the effects of EOs and their MICs and MBCs on pathogenic isolates could differ when fixed on a BC membrane because the amount and spread of oil within the BC matrix vary. Therefore, BC was made into discs and infused with emulsified TEO, as explained in the methods section. Unexpectedly, the BC discs with the original TEO showed no inhibition zone, which means that there was no antibacterial action. Conversely, the BC discs enriched with emulsified TEO had antimicrobial activity. The best inhibition zone was observed at 800 µL/mL, with all the strains showing inhibition zones ranging from 12.7 ± 0.58 to 14.3 ± 2.08 mm (Table 4). Essential oil compounds are known to evaporate quickly when exposed to air at room temperature (Abu-Zeid 1992). This could explain the deficiency of BC discs loaded with the original TEO to exhibit antibacterial activity, since they might have been exposed to evaporation during the incubation of inoculated plates at 37 °C. However, we believe that emulsifying the oil could stop evaporation, facilitate its movement through the BC matrix, and increase its retention, which explains the antibacterial activity observed in bacterial cellulose discs with an oil emulsion compared with the original oil. According to Tables 2, 4, the clear zone around the BC/TEO discs for *K. rosea* was smaller than that around the BC/TEO discs for filter paper discs containing TEO (12.5 mm and 17 mm, respectively). These findings suggest that the BC matrix thickness, surface area, and structure significantly affect the release and inhibitory activity of EOs. These results are consistent with those of Nagmetova et al. (2020), who reported that the inhibitory effect of BC impregnated with oregano (OEO) varies depending on the BC-producing strain due to differences in thickness and fiber arrangement. In addition, Srikandace et al. (2018) concluded that the inhibition zone was lower when citrus essential oils (EOs) were incorporated into bacterial cellulose-based edible films, than when EOs were used alone against *E. coli* and *S. aureus*. However, the inhibition zone for *E. cloacae* increased, from approximately 10 mm with TEO on filter paper to 14 mm with BC/TEO. The varying response could be linked to the different sensitivities of the bacteria to the BC/TEO composite. Terpenes, including D-limonene and γ-terpinene of tangerine (*C. reticulata*) EO, have been reported to exhibit antimicrobial activities against both antibiotic-susceptible and antibiotic-resistant bacteria, via their ability to promote cell rupture and inhibit protein and DNA synthesis (Álvarez-Martínez et al. 2021; Musara et al. 2020). Additionally, D-limonene has shown a wide spectrum of antimicrobial activities, making it a promising antimicrobial agent against food-borne pathogens, especially in combination with ε-polylysine (Chikhoune et al. 2013; Van Vuuren and Viljoen 2007; Zahi et al. 2017).

(c) Application of TEO immobilized on BC as bioactive absorbent pads (BAPs) to achieve microbial safety in fish fillets

The terpenes and terpenoid components of essential oils exhibit antimicrobial activity against foodborne bacteria, and their use as food flavoring agents provides a promising alternative to conventional bactericides and fungicides in the food industry (Perricone et al. 2015; Masyita et al. 2022).

D-limonene is the main compound present in all citrus-derived essential oils (EOs) and is widely used as a flavoring agent in commercial applications such as the food and beverage industry because of its transparency and pleasant citrus fragrance (Zahi et al. 2017). To create effective bioactive absorbent pads (BAPs), the partially dehydrated BC membrane was saturated with emulsified TEO. The optimal amount of emulsified TEO was found to be 200 µL, as this fully saturated the BC without requiring any excess oil removal.

### Effects of BC as a bioactive absorbent pads (BAPs), gamma irradiation and their combination on the log bacterial count in fish fillet as a model food system

Absorbent pads play a critical role in meat packaging, helping prolong shelf life and ensuring meat safety (Ren et al. 2018). Most research on absorbent pads has concentrated on beef and poultry rather than seafood products. Thus, this study sought to investigate how bioactive BC pads (BC/TEO), gamma irradiation, and their combination affect the initial population of four pathogenic strains artificially introduced into fish fillet samples. The fish fillet samples were exposed to 2 kGy of gamma irradiation alone or 1.0 kGy combined with BC or BC + TEO; the results are presented and discussed in Tables 5, 8. The initial microbial count of the four isolates (control) was 5.4 log CFU/g, which increased to 8.8 log CFU/g after six days of storage. However, the use of BC, BC + TEO, BC + (1.0 kGy) of gamma radiation, and BC + TEO + (1.0 kGy) of gamma radiation led to decreases of 1.9, 3.4, 3.4, and 4.9 log CFU/g, respectively, after the same storage period. Compared with the control treatment, these combined treatments significantly (*p* < 0.05) impacted the bacterial population count after six days of storage. This reduction in microbial load may be due to the ability of cellulose to absorb fluids from the fish fillet, which prevents the provision of nutrients for bacterial growth. While cellulose pads effectively absorb fluids, they lack antimicrobial effects (Otoni et al. 2016; Bovi et al. 2018; Agrimonti et al. 2019). The incorporation of food-grade antimicrobial agents can promote their effectiveness against bacterial proliferation (Agrimonti et al. 2019; Mocanu et al. 2019; Li et al. 2021; Zhou et al. 2020; Hu et al. 2025), thereby delaying deterioration, maintaining quality, and improving stability during storage. Hasanin et al. (2025), prepared bioactive nanopackaging films with antioxidant and antimicrobial activity using nanochitosan from shrimp shells, nanocellulose and essential oil extract from the white and orange parts of orange peel waste. Nonetheless, the combined effect of BC food pads and gamma irradiation on fish fillet pathogens has not yet been studied, except for certain studies highlighting the synergistic action of radiation alongside naturally occurring active substances (Table 8). The present results showed that both BC and BC-BAPs could absorb unwanted odors from stored fish samples, which is crucial for consumer satisfaction. Fresh fish often possess a muddy scent due to secondary metabolites such as geosmin, in addition to compounds such as heptanal, hexanal, and volatile organic compounds such as aldehydes, ketones, esters, and sulfur (Deng et al. 2022; Huang et al. 2021). A major obstacle in storing and transporting fish products is controlling their odor, which can diffuse outside the packaging and interact with its material (Kimbuathong et al. 2020). To solve this problem, essential oils with strong and desirable scents, such as lime, ginger, garlic, or oregano, can be incorporated into packaging to reduce the fishy aroma of seafood (Laorenza et al. 2022). Research has shown that carvacrol, citral, and α-terpineol essential oils can be released from the polymer matrix of the packaging into the headspace (Laorenza et al. 2021). Furthermore, adding garlic pickle and tangerine peel to packaging has been found to eliminate the fishy odor of mackerel and hairtail stew (Yoo 2014). In a recent study, BC immobilized by citral (BC-citral) was prepared using gas-phase adsorption technology and successfully used for flavor adsorption, showing promise for the food industry (Hu et al. 2025).

**Table 8:** Effects of the combination of the BC-TEO bioactive system and gamma irradiation on artificially inoculated FFS

Treatment	Microbial population count (CFU/g)		Interpretation	Previous compared studies	
	Day 1 (after 2 h)	Day 6		Explanation	References
Control (without any treatment)	The bacterial count was 5.47, 5.63, 5.14, and 5.25 log CFU/g *for K. oxytoca, S. ficaria, E. cloacae, and K. rosea,* respectively	The growth curve of bacteria generally increases over the storage period. The count was 8.89, 8.91, 8.72, and 8.69 log CFU/g, respectively	The presence of fluids that are excreted from the FFS during storage represents a source of nutrients and a suitable environment for microbial growth and food spoilage	The leftover blood and tissue fluids in chilled meat are the primary factors that contribute to microbial contamination, leading to food-borne illnesses	Hultman et al. (2015)
BC pads	The bacterial count was 5.43, 5.04, 5.07, and 5.07 log CFU/g, respectively, which is lower than the control samples	The bacterial count was reduced in the four pathogenic strains by 1.59, 1.68, 1.92, and 2.35 log CFU/g, respectively	This reduction in microbial load may refer to the ability of BC to absorb fluids and separate them from the fish samples, causing a deficiency of nutrients required for bacterial growth	Absorbent mats inhibit the growth of microorganisms by absorbing the exudate of the chilled meat to form a relatively dry environment, thereby prolonging their shelf life	de Azeredo (2013), Ren et al. (2018), Tian et al. 2023
BC + TEO(Bioactive pads)	The bacterial counts were reduced by 0.98, 1.16, 1.07, and 0.89 log CFU/g, respectively	Compared to control, BC/TEO pads reduced the initial load of the four bacteria by 3.21, 3.12, 3.36, and 3.72 log CFU/g, respectively	The incorporation of TEO in BC functions as bioactive pads, helping to minimize cross-microbial contamination between the FF and the underlying pads. This is achieved through the antimicrobial properties of TEO and the BC pad's ability to absorb excess nutrient-rich exudate from FFS	Incorporation of food-grade antimicrobials into absorbent pads may provide a means to slow down deterioration, maintain quality of meat, and improve stability during storage Incorporation of antibacterial agents like citral oil into BC can effectively inhibit the growth of *E. coli* and* K. rosea* from 2.77% and 5.74% to 80.85% and 89.16%, respectively, and prolong the shelf life of food Cellulosic pads infused with emulsions containing thyme and oregano essential oils demonstrated antimicrobial activity against certain meat bacterial species, and common foodborne pathogens and extended the shelf life of minced meat and hamburgers stored at 4 °C to 16 and 12 days, respectively The major active components of TEO are phenols, terpenes and aldehydes; that act principally against the cell cytoplasmic membrane due to their hydrophobic nature and affecting the unsaturated fatty acid on the bacterial membrane, thus altering its structure	Agrimonti et al. (2019), Mocanu et al. (2019), Li et al. (2021), Zhou et al. (2020), Hu et al. (2025) Hu et al. (2025) Agrimonti et al. (2019) Chandharakool et al. (2020)
gamma irradiation (2.0 kGy)	The gamma irradiation reduced the initial load of pathogenic isolates by 3.24, 3.16, 2.32, and 2.79 log cycles, respectively	The initial load of the four bacterial pathogens was reduced by 4.20, 4.91, 4.12, and 4.76 log cycles, respectively	The current study indicated that gamma irradiation has a strong antibacterial effect against the bacteria under investigation	Gamma- irradiation is a tried-and-true technology that improves the safety, quality, and shelf life of meat products, and it is also an energy-efficient, safe, effective, and eco-friendly process The previous studies proved that gamma irradiation of 2–7 kGy is considered a successful method in fish preservation since it can reduce the populations of food-borne bacterial pathogens as well as many fish-specific bacterial spoilers, and can extend the shelf life of fish	Hashim et al. (2024) Islam, (2025)
BC + gamma irradiation (1.0 kGy)	The pathogenic isolates have decreased to 3.74, 3.81, 3.47, and 3.77 log cycles, respectively	The population counts of tested bacteria decreased by 3.53, 3.26, 3.26, and 3.37 log cycles, respectively	The high reduction of the microbial population was caused by the combined effects of gamma irradiation and BC. Gamma radiation affect the bacterial cell and BC absorbs FF exudates and prevent a nutrient source of bacterial growth	Applying irradiation in conjunction with other techniques has been shown to enhance the effectiveness of reducing adverse effects. By using lower doses, this approach is recommended for controlling microbial contamination in foods and ensuring their quality	Ouattara et al. (2002), Hussain et al. (2016)
BC + TEO + gamma irradiation (1.0 kGy)	The surviving populations have decreased to be 2.32, 2.04, 2.17 and 2.89 log cycles, respectively	The population counts decreased 4.97, 5.02, 4.95, 4.83, respectively	The combination of BC, TEO, and gamma radiation had the most significant effect, resulting in a markedly lower overall bacterial count of all pathogenic strains BC soaks up fluids from FF and does not provide much nutrients for bacteria to grow. Gamma radiation and harm bacteria by changing their cell wall permeability, causing cell rupture, or affect the protein and DNA synthesis	-The combined effects of gamma irradiation at 2.0 kGy, and active packaging (PLA/lemongrass and cumin essential oils (PLA/mix oil)) enhanced the radio-sensitivity *of Salmonella enteritidis, E. coli, and Listeria monocytogenes* inoculated in poultry meat Astaxanthin, a shrimp waste recovery agent, combined with gamma irradiation (1.5 kGy), demonstrated that the minced fish samples retained their microbiologic acceptability for up to 20 days, compared to 7 days for the control - During the eight-week storage period, *A. flavus* and* A. parasiticus*, which were inoculated separately in sorghum and peanut, respectively, were successfully eradicated by the combination of low-dose gamma -irradiation (2.0 kGy) and star anise essential oil at a concentration of 0.5 μL/g^−1^ -Bacterial cells are primarily harmed by gamma irradiation through the disruption of chemical bonds in their DNA, alterations in membrane permeability, and changes to cellular functions. These modifications can enhance the sensitivity of bacteria to antimicrobial agents by improving the interaction between antimicrobial compounds and the cell membranes	Abdel Khalek et al. (2022) (El-Bialy and Abd El-Khalek 2020) Abdel Khalek et al. 2021 Lopez-Gonzalez et al. (1999)

### Potential mode of action of the combined bioactive system on the FF model system

**Fig. 5:**
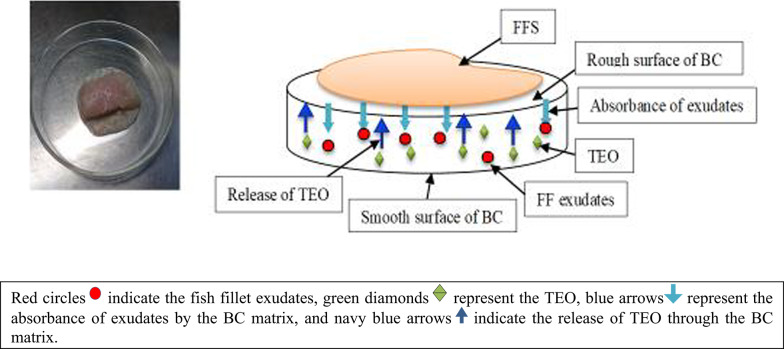
Graphical visualization of the potential mode of action of the combined bioactive system on artificially inoculated fish fillet samples as a model food system

The mechanism of the current bioactive system, as indicated in Table 9 and Fig. 5, can be determined on the basis of the obtained results and on the basis of the previous findings.

**Table 9:** Potential mode of action of the combined bioactive system (BC-bioactive pads and gamma irradiation) on artificially inoculated fish fillet samples as a model food system

Combined bioactive system (CBS)			
Consists of	Bioactive absorbent pad (BAPs)		Gamma irradiation
	BC	TEO	
Description	Superabsorbent hydrogel	Volatile oil that contain a variety of secondary metabolites (limonene, γ-terpinene, paracymene, β-myrcene, β-pinene, α-pinene.)	^60^Co Gamma Cell GC 220, Canada Co. Ltd. NCRRT
	Three-dimensional (3D) network		Low doses (1–2 kGy)
	Nanofibrils/nanoporous sponge-like structure		An essential tool in food preservation
Mode of action of each item in the bioactive system	Absorption of excessive exudates that are secreted from FFS during storage and keeping it inside the BC matrix, away from contact with FFS, to prevent the growth of pathogens	Acts as a volatile antimicrobial agent that migrates from the BC matrix to the food and helps in creating an environment saturated with antimicrobial agent inside the package	Gamma irradiation may cause: the breakdown of the chemical bonds of DNA, alteration of membrane permeability and cell function, that makes it easier for antimicrobial compounds to come into contact with cell membranes and increasing the sensitivity of bacteria to antimicrobial agents
	Adsorption of undesirable odor produced from FFS during storage	Inhibition of cell wall, protein, and nucleic acid synthesis	
	The BC serves as an effective carrier for antimicrobial materials due to its ability to bind with electropositive metal transition atoms through electrostatic interactions (Fahma et al. 2014; Zhang et al. 2017)	Disruption of cell membrane functionality and increasing its permeability, leading to ion/molecule leakage and death	
	Its nonporous structure facilitates the incorporation and maintenance of TEO within its matrix and controlling its migration and release		
Synergistic effect of BC and TEO	The BC nanostructure immobilized by TEO was acting together in a closed sorption system that ensured the removal of the nutrient source and inhibited the growth of pathogens in a synergistic/spontaneous process. It can retain the TEO within the layers of the BC matrix, ensure the continuity of EO release and its bioactivity, and at the same time absorb the excess secreted liquids like water, blood, and other fluids that promote microbial growth		
Synergistic effect of TEO and gamma radiation		Based on the hurdle technology approach, combinations of gamma radiation with other antimicrobial agents, such as essential oils, were more effective against microorganisms. The efficiency of FFS decontamination was improved due to their synergistic effects, which means that the BC/TEO pads increased the radio-sensitivity of tested bacteria	
Whole mechanism of BC, TEO and gamma radiation	The combination of BC with its nanostructure, TEO, and gamma irradiation with their antimicrobial properties improves the efficiency of FFS decontamination through their synergistic effects and the additional lethal damage caused by the interaction of all agents, as previously described. Furthermore, the system may act in a spontaneous and/or subsequent mechanism, where the absorbed drips substitute the evaporated TEO and/or vice versa, where the absorbed drips initiate the release of TEO in a sophisticated mechanism		
Other factors affecting the activity of the system	**The morphological characteristics of BC**	**Tween 80**	**The low storage temperature**
	The BC-BAP contains two faces: one is downward, which is smooth and prevents the drips from going out of the pads. While the other side is rough and upward in direct contact with the FFS to simplify the absorption of the undesirable fluids and at the same time allow the release of TEO into the package	Tween 80 was used as an emulsifying agent of TEO, as it has a basic structure of a hydrophilic head bearing charges that are soluble in water and a long lipophilic tail, which is soluble in fat (Das et al. 2024)	The low storage temperature (4 °C) contributed to the preservation of FFS and, at the same time to controlling the release of TEO by maintaining it for a long time without complete evaporation
		It may reinforce the bioactive system by bound TEO evaporation and controlling its release. In addition to inhibiting microbial adhesion and preventing biofilm formation (Allegrone et al. 2021)	

### Characterization of TEO using GC‒MS

GC/MS is a precise and versatile method for analyzing volatile compounds of essential oils. The results (Table 6 & Fig. 4) revealed that the main compounds were D-limonene (79.9%) and γ-terpinene (11.9%), which agreed with the findings of a previous study reporting that the major compounds of tangerine oil are D-limonene and γ-terpinene (74.47% and 10.86%, respectively) (Chandharakool et al. 2020). In contrast, α-pinene (1.4%), ß-myrcene (1.3%), methyl methanthranilate (1.1%), and L-α-terpineol (1.0%) represented a minor portion of the total compounds and differed from those reported in previous studies because of variations in the planting conditions, including the soil geochemistry, season of harvesting, and method of extraction. Eneke et al. (2021) reported that GCMS analysis of the dichloromethane extract of tangerine peel revealed the presence of 16 bioactive compounds, where n-hexadecanoic acid was present at the highest concentration (26.400%), followed by D-limonene, 9,12-octadecadienoic acid (Z, Z)-, and oleic acid (23.049%, 18.849%, and 7.272%, respectively). Another study involving TEO analysis (Bhatia et al. 2024) identified approximately 40 distinct chemical compounds, including limonene (43.85%), linalyl acetate (19.16%), linalool (18.38%), and ß-myrcene (3.41%). Song et al. (2024) reported that tangerines could be differentiated from other citrus fruits (mandarins, sweet oranges, and hybrids) by their elevated volatile oil extraction rates and higher levels of compounds such as O-cymene, α-terpinene, d-α-pinene, terpinolene, γ-terpinene, l-β-pinene, and 3-thujene.

#### Characterization of BC and BC/TEO samples via FTIR and GC/MS analysis

Owing to the complexity of the correlation between FTIR and GC/MS analysis, the determination of the functional groups of the compounds identified by GC/MS through the location of their different peaks in the FTIR spectrum is often limited. Thus, the differentiation among the BC and BC/TEO samples in both the irradiated and non-irradiated states was illustrated.

Generally, the main characteristic groups of BC in FTIR spectroscopy are located at wavelengths of 3500 cm^−1^ (OH stretching), 2900 cm^−1^ (CH stretching of alkanes and asymmetric CH₂ stretching), 2700 cm^−1^ (CH₂ symmetric stretching), 1640 cm^−1^ (OH deformation), 1400 cm^−1^ (CH₂ deformation), 1370 cm^−1^ (CH₃ deformation), 1340 cm^−1^ (OH deformation), and CO deformation at 1320–1030 cm^−1^. The bands in the region of 1000–1200 cm^−1^ are -associated- with stretching C–O–C and C–O vibrations, and the peak at 847 cm^−1^ characterizes β-1,4 bond vibrations (Zhbanko 1966 and Atykyan et al. 2020). The FTIR analysis of the control BC samples (Table 7 and Fig. 7 [SM1]) revealed characteristic peaks at 2900.7 cm^−1^ (C–H stretching of alkane and asymmetric CH₂), 2721.8 cm^−1^ (–CH₂ symmetric stretching, which disappeared in BC/TEO), and deformation peaks for the –OH, CH₂, and CH₃ groups at approximately 1572–1586 cm^−1^, 1422–1458 cm^−1^, and 1365.7 cm^−1^, respectively. There were no significant changes between the BC samples before and after irradiation at a dose of 1.0 kGy except in the –OH region, where a shift (from 3290.9 and 3335.9 to 3214.6 cm^−1^, respectively) occurred due to the interactions among the free radicals formed resulting from irradiation. However, significant spectral changes were observed after impregnation with tangerine essential oil (TEO), especially in the –OH region.

**Fig. 6:**
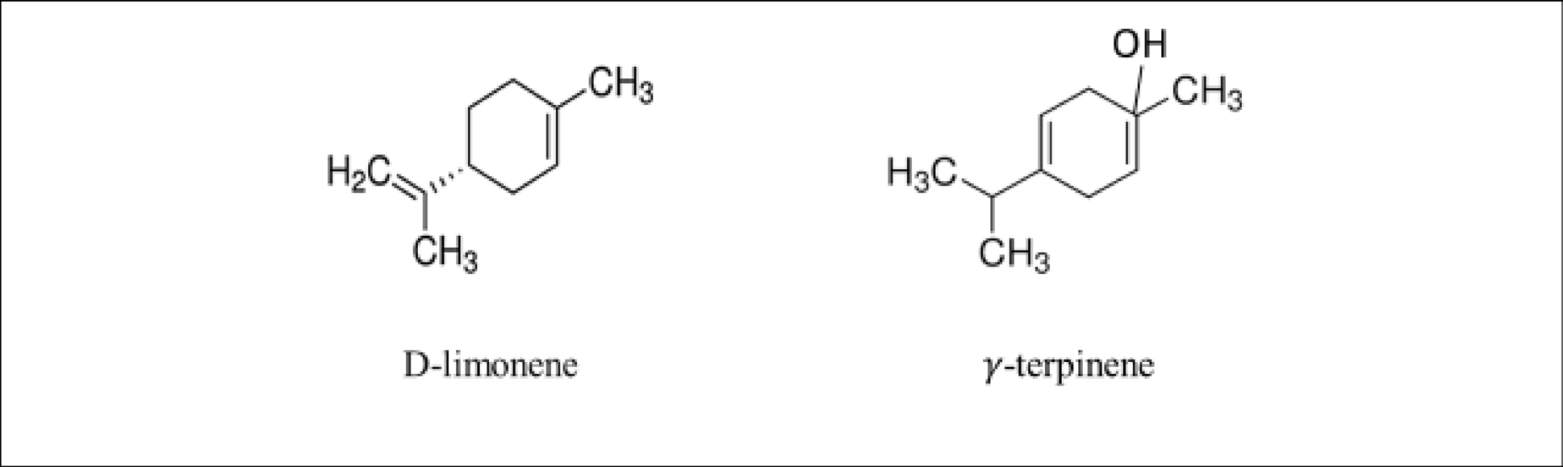
Molecular structures of D-limonene and γ-terpinene. https://www.sigmaaldrich.com/EG/en

According to the results of the GC/MS analysis, the main components of TEO are limonene and terpinene, which are simple hydrocarbon structures (Perveen 2018) with a chemical formula of (C_10_H_16_). As shown in Fig. 6, D-limonene consists of a cyclohexene ring attached to a methyl and an isopropyl group, whereas γ-terpinene consists of a cyclohexadiene ring (a six-membered carbon ring with two double bonds) substituted with methyl and isopropyl groups (Unsal et al. 2022**).** The GC/MS analysis of the TEO oil was performed by Bhatia and colleagues, who identified approximately 40 distinct chemical compounds, whereas the FTIR spectrum indicates the interactions among the functional groups of the pectin- and gelatin-based edible film components (Bhatia et al. 2024).

On the basis of previous studies, the broad –OH bending region at 3425.8–3558.8 cm^−1^ corresponds to alcoholic compounds (linalool, terpinen-4-ol, α-terpineol, and octanal) in TEO (Elzey et al. 2016). The peaks at 2826.2–2941.9 cm^−1^ reflect asymmetric C–H stretching vibrations from methyl (–CH_3_) and methylene (–CH_2_) groups in limonene and terpinene, where the broad intensity of this band in irradiated BC/TEO suggests enhanced cross-linking (Ahmed et al. 2014). On the other hand, the intense peak at 1734–1732 cm^−1^ indicates –C=O groups, such as aldehydes and esters, related to carbonyl group deformation vibrations in TEO components, whereas the peak at 1586–1588 cm^−1^ marks the fingerprint region for citrus oil, indicating unsaturated aromatic compounds (limonene and terpinene) and aromatic nuclei vibrations (Magnago et al. 2020). However, the peak at 1458 cm^−1^ corresponds to C–H doublet stretching in the methylene groups of aromatic compounds in TEO (Flores et al. 2024). However, the absence of the expected C=C bond stretching peak (1600–1680 cm^−1^) suggests possible cleavage of aromatic rings in the terpenes after impregnation and irradiation. Recently, the adsorption mechanism of citral on BC was proven to involve spontaneous adsorption through van der Waals forces and hydrogen bond interactions. This mechanism results in a stable BC‒Citral composite material used in controlled-release applications, such as in the food industry, owing to its ability to delay the release of citral and provide antibacterial properties (Hu et al. 2025).

## Conclusion

The present study provides important data for assessing and managing the risk associated with the presence of emerging foodborne pathogenic bacteria in fish fillets. This research promotes the development of effective bioactive systems from renewable bioresources and sustainable biomaterials as an innovative strategy for food preservation. The findings successfully monitored and controlled the bacterial load of four newly emerged pathogens isolated from fish fillet samples. The use of biomaterials (bacterial cellulose), physical nonthermal methods (gamma radiation), and natural preservatives extracted from plants (tangerine essential oils) as a bioactive system, which serve as functional, sustainable, and eco-friendly absorbent pads for reducing pathogenic and spoilage bacteria in fish fillet samples, has been achieved. BC contains nanofibrils and a nanoporous structure that enhances the efficiency and functionality of the system. This formulation releases active substances that inhibit microbial growth, absorbs excess fluids excreted from fish samples, and eliminates undesirable odors during storage. Compared with the individual treatments, the combination of gamma irradiation with active pads enhanced antibacterial effectiveness. This study allows further research on newly emerged bacterial strains contaminating seafood products, potentially highlighting pollution problems or reflecting the impact of climate change.

## Future work

Future research should focus on studying the synergistic effects of multiple essential oils as antimicrobial agents in food pads. This approach aims to increase their efficacy in reducing microbial contamination. Crucially, the toxicity of the developed bioactive system must be assessed, and sensory evaluations should be performed to ensure its usability and safety. Furthermore, studying the mechanism of the BC-bioactive system is essential for optimizing its performance and functionality.

## Supplementary Information

Below is the link to the electronic supplementary material.

Supplementary Material 1.

## Data Availability

All the data were included in the manuscript and the FTIR spectra (Fig. 7) were attached as supplementary information [SM:1].

## Abbreviations

BC: Bacterial cellulose
FDA: Food and Drug Administration
GRS: Generally, recognized as safe
TEO: Tangerine essential oil
MEO: Mandarin essential oil
LEO: Lemon essential oil
HS: Hestrin-Schramm
SW: Sugared-water
kGy: Kilo gray
FFS: Fish fillet samples
MIC: Minimum inhibitory concentration
MBC: Minimum bactericidal concentration
SCOBY: Symbiotic culture of bacteria and yeast
BC-BAPs: BC bioactive absorbent pads
MFS: Model food system
CFU: Colony forming unit
PCA: Plate count agar
