# Supplementary material for: Combinational effect of gamma irradiation and sustainable bioactive absorbent bacterial cellulose pads impregnated with tangerine essential oil against some bacterial fish fillet-borne pathogens
Source: Bioresour Bioprocess. 2026 May 5;13(1):65. doi: 10.1186/s40643-026-01061-0 (PMC13144468; doi:10.1186/s40643-026-01061-0)
Supplement: Supplementary file 1 — Supplementary Material 1. [file 40643_2026_1061_MOESM1_ESM.docx]

**Fig. 7 FTIR spectra of: a) BC- control, b) BC after irradiation (1.0 kGy),**

**c) BC impregnated with TEO, d) BC after impregnation with TEO and irradiated (1.0 kGy)**

1. **(b)**

**(c) (d)**
